# Cognitive Trajectories in Community-Dwelling Older Adults and Incident Dementia, Disability and Death: A 10-Year Longitudinal Study

**DOI:** 10.3389/fmed.2022.917254

**Published:** 2022-06-27

**Authors:** Zimu Wu, Robyn L. Woods, Trevor T. J. Chong, Suzanne G. Orchard, Raj C. Shah, Rory Wolfe, Elsdon Storey, Kerry M. Sheets, Anne M. Murray, John J. McNeil, Joanne Ryan

**Affiliations:** ^1^School of Public Health and Preventive Medicine, Monash University, Melbourne, VIC, Australia; ^2^Turner Institute for Brain and Mental Health, Monash University, Melbourne, VIC, Australia; ^3^Department of Neurology, Alfred Health, Melbourne, VIC, Australia; ^4^Department of Clinical Neurosciences, St Vincent's Hospital, Melbourne, VIC, Australia; ^5^Department of Family Medicine and Rush Alzheimer's Disease Center, Rush University Medical Center, Chicago, IL, United States; ^6^Division of Geriatrics and Palliative Medicine, Department of Medicine, Hennepin Healthcare, Minneapolis, MN, United States; ^7^Berman Center for Outcomes and Clinical Research, Hennepin Healthcare Research Institute, Minneapolis, MN, United States

**Keywords:** aging, cognition, dementia, activities of daily living, death, longitudinal

## Abstract

**Objective:**

The inter-individual variability in cognitive changes may be early indicators of major health events. We aimed to determine whether late-life cognitive trajectories were associated with incident dementia, persistent physical disability and all-cause mortality.

**Methods:**

Data came from a cohort of older community-dwelling individuals aged 70 years or above in Australia and the United States. Global cognition, verbal fluency, episodic memory and psychomotor speed were assessed regularly at up to seven waves between 2010 and 2017. Dementia, disability in activities of daily living, and death were adjudicated between 2017 and 2020. Latent classes of cognitive trajectories over seven years were determined using group-based trajectory modeling. Multivariable logistic regression was used for the prospective associations between cognitive trajectories and these outcomes.

**Results:**

Cognitive trajectories were defined for 16,174 participants (mean age: 78.9 years; 56.7% female) who were alive and without incident dementia or disability by 2017, among which 14,655 participants were included in the association analysis. Between three and five trajectory classes were identified depending on the cognitive test. Cognitive trajectories were strongly associated with the risk of dementia. For example, compared to those in the highest-functioning trajectory, the worst performers of episodic memory had a 37-fold increased risk of dementia (95% CI: 17.23–82.64). The lowest trajectories of both global cognition and episodic memory also predicted increased mortality risk (OR: 1.80, 95% CI: 1.28–2.52; OR: 1.61, 95% CI: 1.09–2.36, respectively), while only slow psychomotor speed was marginally associated with physical disability (OR: 2.39, 95% CI: 0.99–5.77).

**Conclusions:**

In older individuals, cognitive trajectories appear to be early indicators of clinically relevant health outcomes. Systematic cognitive assessments as part of routine geriatric evaluation may facilitate early identification and interventions for those individuals at highest risk.

## Introduction

Maintaining good cognitive function is an important component of healthy aging (1, 2). There is heterogeneity in late-life cognitive aging across individuals, with distinct cognitive trajectories observed in older populations (3). Gradual decline in cognitive function at old age has been thought to be a ‘normal' part of the aging process (4). However, some individuals experience more advanced cognitive decline dementia (5), while others appear to sustain high cognitive function over time even with advancing age (6).

A decline in global cognition and memory is associated with an increased risk of brain atrophy and dementia (7, 8), and some preliminary evidence suggests that worse cognitive trajectories also predict the risk of other geriatric outcomes (3). For example, one study observed that lower trajectories in global cognition were associated with a higher burden of hospitalization (9), and another two studies reported that declining global cognitive trajectories increased the incidence risk of physical disability and mortality (10, 11). However, most previous studies only examined global cognition, often using the Mini-Mental State Examination, which is a relatively crude measure (9, 12–15). It is unknown whether changes in specific domains of cognitive function are early indicators of important health outcomes.

This study aimed to investigate the relationship between global and domain-specific trajectories of cognitive function, and the incidence risk of dementia, persistent physical disability and mortality. We also examined the association between cognitive trajectories and a composite measure of these three outcomes - disability-free survival that reflects a healthy lifespan. Using data collected from a large cohort of older adults, the findings disentangle the population heterogeneity of late-life cognitive trajectories and demonstrate how these can be used as an early marker for major health outcomes.

## Materials and Methods

### Study Sample

Study participants came from the ASPREE (ASPirin in Reducing Events in the Elderly) clinical trial – a randomized placebo-controlled trial that investigated the long-term effects of daily low-dose aspirin on health outcomes of older adults (16). A total of 19,114 participants aged 65+ years (African American and Hispanic/Latino) and 70+ years (Australian White, American White and all other ethnicities) were recruited from Australia and the United States between March 2010 and December 2014. At enrolment, eligible participants were required to be without a diagnosis of dementia and scored over 77 in the Modified Mini-Mental State Examination (3MS). Additionally, participants were without any severe difficulty performing any of the basic activities of daily living (ADL), established cardiovascular diseases, or any life-threatening illness (17).

### Assessment of Cognitive Function

Cognitive function was assessed at baseline and annually over follow-up, at up to six waves. The battery included 1) 3MS for global cognitive function (18), 2) single-letter (F) Controlled Oral Word Association Test (COWAT-F) for verbal fluency (19), 3) Hopkins Verbal Learning Test-Revised delayed recall task (HVLT-R) for episodic memory (20), and 4) Symbol-Digit Modalities Test (SDMT) for psychomotor speed (21). In addition, an overall score was generated by adding up the z-scores of the four cognitive tests (6, 22).

### Ascertainment of Study Endpoints

The endpoints of this study included dementia, persistent physical disability, all-cause mortality and disability-free survival. Dementia was adjudicated according to the criteria of the Diagnostic and Statistical Manual of Mental Disorders, fourth edition (23). This was done in conjunction with several evaluations including Alzheimer's Disease Assessment Scale–Cognitive subscale (24), Color Trails (25), Lurian overlapping figures (26), and the Alzheimer's Disease Cooperative Study Activities of Daily Living scale (27), as well as other relevant information such as laboratory tests, neuroimaging tests, clinical case notes and hospital medical records. Persistent physical disability was defined as the inability to perform or severe difficulty in performing one or more basic ADLs (28). Persistence was determined as the loss of the same ADL for at least 6 months, or adjudicated admission to nursing care facility due to physical disability (17). Death was confirmed upon verification with at least two independent sources (e.g., family or Primary Care Partnerships report, or clinical record, or public death notice) (17), with linkage to the National Death Index (17). Disability-free survival, which is a composite endpoint analyzed as the primary outcome in the ASPREE clinical trial, was defined as the first occurrence of dementia or persistent physical disability or all-cause mortality, whichever came first (29).

### Statistical Analysis

Group-based trajectory modeling (GBTM) was used to identify the latent classes of cognitive trajectory, for each cognitive test. GBTM is an application of finite mixture modeling, which captures the underlying subgroups based on the longitudinal patterns of an indicator (30). The goodness of fit and model adequacy was used to select the best model (30, 31) (details in Supplementary Material). Individuals were required to have cognitive data at baseline and at one or more subsequent visits, to be eligible for trajectory modeling.

To ensure temporality, cognitive trajectories were modeled over the trial period (from March 2010 until June 2017), and incident health outcomes were examined between June 2017 and November 2020. As such, participants included in this analysis needed to be alive at the end of the trial period, and without dementia or physical disability.

Basic characteristics were presented as numbers and proportions, and compared between subgroups using chi-squared test. Multivariable logistic regression was used to analyze the association between cognitive trajectories and subsequent health outcomes, using the highest-functioning trajectory class as the reference group. The models adjusted for self-reported socio-demographic characteristics including age at the last visit (continuous), gender (men, women), ethnicity (Australian White, African American, Hispanic/Latino, US White, others) and education (≤12 years, >12 years), as well as smoking status (never, former, current), alcohol intake (never, former, current), living situation (at home alone, at home with someone or in a residential home), body mass index (underweight or normal, overweight, obese), hypertension (no, yes - defined as being on treatment for high BP or BP >140/90 mmHg), diabetes mellitus (no, yes - defined as being on treatment for diabetes, or fasting glucose≥126 mg/dL (≥7 mmol/L), or self-report), dyslipidemia (no, yes - defined as taking cholesterol-lowering medications, or serum cholesterol ≥212 mg/dL (≥5 mmol/L; Australia) and≥240 mg/dL (≥6.2 mmol/L) or LDL >160 mg/dL (>4.1 mmol/L)), depression (no, yes - defined as a score of 8 or higher in the 10-item Center for Epidemiological Studies-Depression Scale), frailty index (continuous, constructed based on 67 health deficits) (32), weak grip strength (no, yes - defined as grip strength in the lowest 20% at baseline adjusted by gender and BMI) (33), slow gait speed (no, yes - defined as gait speed in the lowest 20% at baseline adjusted by gender and height) (33). Grip strength and gait speed were not included in the models for persistent physical disability, as they are closely related to this dependent variable. The information of all covariates was ascertained at baseline (gender, ethnicity and education) or the last study visit when cognitive function was assessed.

All statistical analyses were conducted between September 2021 and February 2022, using Stata version 16.0 (Stata Corp., College Station, Texas, USA) and the ‘Proc Traj' package, with statistical significance set as a two-sided *p*-value < 0.05.

## Results

### Identification of Cognitive Trajectories

**Figure 1:**
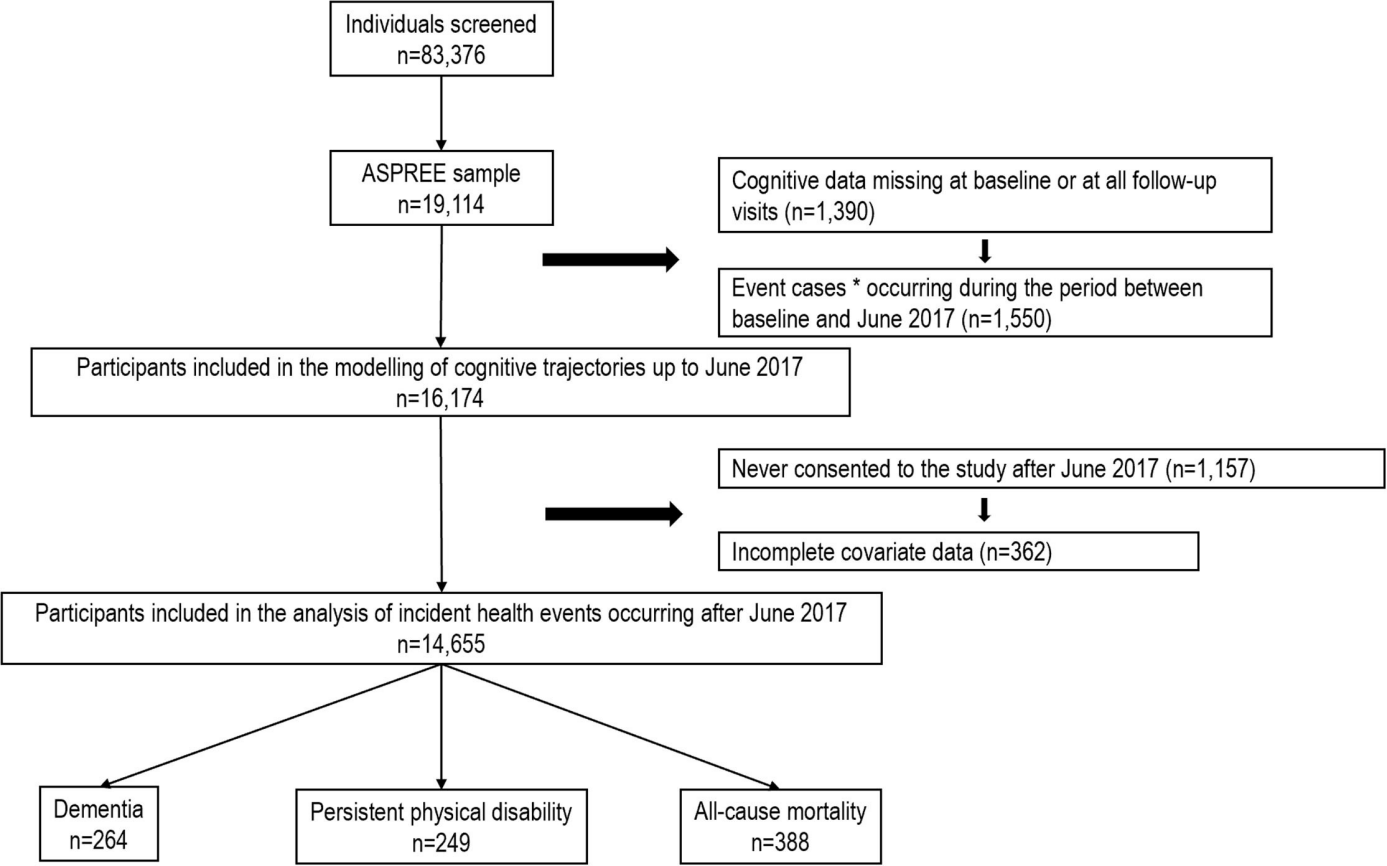
Flow diagram of participants included in the analysis. *Incident dementia, persistent physical disability and all-cause mortality.

**Figure 2:**
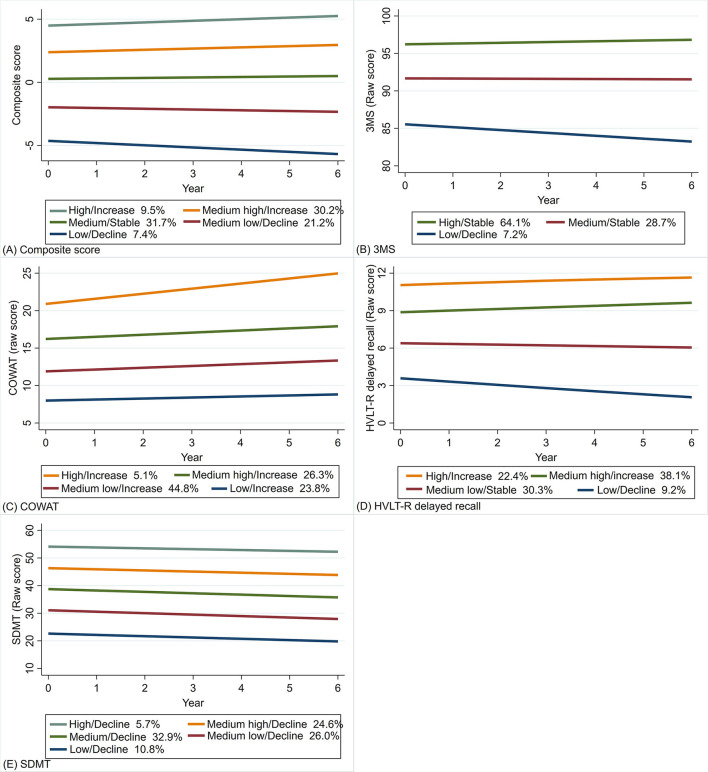
Trajectory plots of the composite score **(A)**, and the raw scores of 3MS **(B)**, COWAT-F **(C)**, HVLT-R delayed recall **(D)** and SDMT **(E)** (*N* = 16,174). (1) the x-axis denotes the year of cognitive assessment at baseline as well as 1, 3, 4, 5 and 6 years of follow-up; (2) the y-axis denotes the composite score and raw scores of the four cognitive tests; (3) the percentages refer to the proportions of participants assigned into the corresponding classes. 3MS, Modified Mini-Mental State Examination; COWAT-F, Controlled Oral Word Association Test-F; HVLT-R, Hopkins Verbal Learning Test–Revised (delayed recall); SDMT, Symbol Digit Modalities Test.

A total of 16,174 individuals were included in the trajectory modeling (Figure 1). For most participants, data on all cognitive tests were available at three or more timepoints (Supplementary Tables 1–3). Between three and five trajectory classes were identified depending on the cognitive test (Figure 2, Supplementary Table 4).

The model identified five classes for the overall score, with higher classes having higher baseline scores and less decline (Figure 2A). For the 3MS test, three classes were identified (Figure 2B), including one class with the highest baseline and least decline that accounted for most participants (64.1%). The four classes identified for COWAT-F (Figure 2C) mainly differed in baseline scores, but all improved over time with varying degrees. Four classes were identified for HVLT-R delayed recall (Figure 2D). The higher two trajectories with higher baseline scores showed slight improvement, while the lower two declined over time. In terms of SDMT, the five classes were crudely parallel, and all showed minor decline at a similar rate (Figure 2E).

### Cognitive Trajectories and Associated Health Outcomes

Of the 14,655 participants with complete covariate data and who were alive and without dementia and physical disability by June 2017, a total of 854 participants reached the endpoints during the post-trial phase. This includes 265 incident cases of dementia, 253 cases of incident physical disability and 568 deaths (Figure 1). Compared to those included in the current analysis, those excluded due to missing data were older, with fewer years of education, and more likely to have chronic conditions (Supplementary Table 5).

Table 1 shows the basic characteristics of participants according to trajectory classes of the overall score. In general, participants in lower-functioning classes were older, more likely to be males, and with notably fewer years of education. They were also more likely to live alone at home, be a current smoker, and never drink alcohol, although the differences were less marked. In addition, all comorbidities were more prevalent in lower-functioning classes, except dyslipidemia which had an opposite trend.

**Table 1:** Basic characteristics of included participants at their last attended follow-up visit (*n* = 14,655).

	**Trajectory classes of the overall score^a^** **No. (%)**					
**Baseline characteristics**	**High/Increase** **(1,454, 9.9%)**	**Medium high/Increase** **(4,558, 31.0%)**	**Medium/Stable** **(4,682, 32.0%)**	**Medium low/Decline** **(2,984, 20.4%)**	**Low/Decline** **(977, 6.7%)**	***P*-value^b^**
**Age, years**						<0.001
65–69^c^	11 (0.8)	14 (0.6)	31 (0.7)	13 (0.4)	1 (0.1)	
70–74	331 (22.1)	828 (18.2)	803 (17.2)	423 (14.2)	86 (8.8)	
75–79	839 (57.7)	2,458 (53.9)	2,224 (47.5)	1,259 (42.2)	344 (35.2)	
80–84	216 (15.0)	927 (20.3)	1,152 (24.6)	787 (26.4)	308 (31.5)	
≥85	67 (4.6)	320 (7.0)	472 (10.1)	502 (16.8)	238 (24.4)	
**Gender**						<0.001
Men	381 (26.2)	1,609 (35.3)	2,244 (47.9)	1,603 (53.7)	609 (62.3)	
Women	1,073 (73.8)	2,949 (64.7)	2,438 (52.1)	1,381 (46.3)	368 (37.7)	
**Ethnicity**						<0.001
Australian white	1,273 (87.6)	4,008 (87.9)	4,184 (89.4)	2,650 (88.8)	894 (91.5)	
African American	16 (1.1)	103 (2.3)	165 (3.5)	124 (4.2)	35 (3.6)	
Hispanic/Latino	21 (1.4)	70 (1.5)	98 (2.1)	88 (3.0)	22 (2.3)	
US white	129 (8.9)	330 (7.2)	171 (3.7)	71 (2.4)	8 (0.8)	
Other^d^	15 (1.0)	47 (1.0)	64 (1.4)	51 (1.7)	18 (1.8)	
**Education, years**						<0.001
<12	417 (28.7)	2,052 (45.0)	2,884 (61.6)	2,171 (72.8)	799 (81.8)	
≥12	1,037 (71.3)	2,506 (55.0)	1,798 (38.4)	813 (27.1)	178 (18.2)	
**Living situation**						0.02
Alone at home	523 (36.0)	1,542 (33.8)	1,604 (34.3)	1,105 (37.0)	363 (37.2)	
With someone	931 (64.0)	3,016 (66.2)	3,078 (65.7)	1,879 (63.0)	614 (62.9)	
**Smoking status**						<0.001
Current	28 (1.9)	74 (1.6)	126 (2.7)	92 (3.1)	35 (3.6)	
Former	560 (38.5)	1,833 (40.2)	1,908 (40.8)	1,290 (43.2)	427 (43.7)	
Never	866 (59.6)	2,651 (58.2)	2,648 (56.6)	1,602 (53.7)	515 (52.7)	
**Alcohol intake**						<0.001
Current-high risk	396 (27.2)	1,194 (26.2)	1,211 (25.9)	711 (23.8)	238 (24.4)	
Current-low risk	742 (51.0)	2,138 (46.9)	2,118 (45.2)	1,202 (40.3)	365 (37.4)	
Former	170 (11.7)	615 (13.5)	681 (14.6)	516 (17.3)	179 (18.3)	
Never	146 (10.0)	611 (13.4)	672 (14.4)	555 (18.6)	195 (20.0)	
**Hypertension^e^**						<0.001
Yes	1,002 (68.9)	3,358 (73.7)	3,656 (78.1)	2,389 (80.1)	782 (80.0)	
No	452 (31.1)	1,200 (26.3)	1,026 (21.9)	595 (19.9)	195 (20.0)	
**Diabetes mellitus^f^**						<0.001
Yes	82 (5.6)	381 (8.4)	456 (9.7)	407 (13.6)	154 (15.8)	
No	1,372 (94.4)	4,177 (91.6)	4,226 (90.3)	2,577 (86.4)	823 (84.3)	
**Dyslipidemia^g^**						<0.001
Yes	1,010 (69.5)	3,028 (66.4)	3,031 (64.7)	1,843 (61.8)	604 (61.8)	
No	444 (30.5)	1,530 (33.6)	1,651 (35.3)	1,141 (38.2)	373 (38.2)	
**Depression^h^**						<0.001
Yes	184 (12.6)	716 (15.7)	863 (18.4)	662 (22.2)	234 (24.0)	
No	1,270 (87.4)	3,842 (84.3)	3,819 (81.6)	2,322 (77.8)	743 (76.0)	
**Weak grip strength^i^**						<0.001
Yes	238 (16.4)	930 (20.4)	1,276 (27.3)	995 (33.3)	409 (41.9)	
No	1,216 (83.6)	3,628 (79.6)	3,406 (72.7)	1,989 (66.7)	568 (58.1)	
**Slow gait speed^i^**						<0.001
Yes	170 (11.7)	892 (19.6)	1,281 (27.4)	1,065 (35.7)	442 (45.2)	
No	1,284 (88.3)	3,666 (80.4)	3,401 (72.6)	1,919 (64.3)	535 (54.8)	
**Body mass index^j^**						<0.001
Underweight/Normal	537 (36.9)	1,387 (30.4)	1,300 (27.8)	842 (28.2)	269 (27.5)	
Overweight	606 (41.7)	1,993 (43.7)	2,090 (44.6)	1,318 (44.2)	448 (45.9)	
Obese	311 (21.4)	1,178 (25.8)	1,292 (27.6)	824 (27.6)	260 (26.6)	
**Frailty index^k^**						<0.001
Frail	119 (8.2)	512 (11.2)	680 (14.5)	544 (18.2)	213 (21.8)	
Pre-frail	464 (31.9)	1,733 (38.0)	1,918 (41.0)	1,290 (43.2)	448 (45.9)	
None-frail	871 (59.9)	2,313 (50.8)	2,084 (44.5)	1,150 (38.5)	316 (32.3)	

a *Overall score was defined as the sum of the z-scores of four cognitive tests including Modified Mini-Mental State Examination, Controlled Oral Word Association Test, Hopkins Verbal Learning Test–Revised (delayed recall) and Symbol Digit Modalities Test*.

b *P-values are based on Pearson's chi-squared test or Fisher's exact test*.

c *Only includes U.S. African American or Hispanic/Latino participants, who were eligible to enroll from 65 years or above (all other participants needed to be 70 years or above to be recruited)*.

d *“Other” was defined as any ethnical category with <100 participants, including Aboriginal/Torres Strait Islanders, American Indians, Native Hawaiian/Pacific Islander/Maori, Asian, more than one race, and those whose ethnicity could not be determined (16)*.

e *Hypertension was defined as on treatment for high BP or BP >140/90 mmHg at study entry*.

f *Diabetes was defined from self-report or fasting glucose ≥126 mg/dL (≥7 mmol/L) or on treatment for diabetes*.

g *Dyslipidemia was defined as those taking cholesterol-lowering medications or serum cholesterol ≥212 mg/dL (≥5 mmol/L; Australia) and ≥240 mg/dL (≥6.2 mmol/L; U.S.) or LDL > 160 mg/dL (>4.1 mmol/L)*.

h *Depression was defined as CES-D-10 ≥8*.

i *Weak grip strength and slow gait speed were defined using the adapted Fried frailty criteria (33)*.

j *Overweight was defined as body mass index ≥25 kg/m^2^ and obesity as body mass index ≥30 kg/m^2^*.

k *Frailty index (range: 0–1) was constructed by 67 deficits and categorized using the cut-off points of >0.21 for ‘frail', and >0.10 and ≤0.21 for ‘pre-frail' (32)*.

The fully adjusted associations of cognitive trajectories with incident dementia and physical disability are shown in Table 2. Compared to the individuals in the highest-functioning trajectory, worse performers with lower baseline scores and/or faster rates of decline showed higher dementia risk in all cognitive tests, although this was less marked for COWAT-F. However, only the lowest class - Low/Decline trajectory of SDMT showed a marginally significant association with physical disability (OR: 2.39, 95% CI: 0.99–5.77, *p* = 0.05).

**Table 2:** Fully adjusted ^a^ associations of cognitive trajectories with incident dementia and persistent physical disability (*n* = 14,655).

	**Dementia^b^**		**Persistent physical disability^c^**	
	**OR (95% CI)**	***P*-value**	**OR (95% CI)**	***P*-value**
**Overall score^d^** **(*****n*****, %)**				
High/Increase (1,454, 9.9%)	Reference		Reference	
Medium high/Increase (4,558, 31.0%)	6.64 (0.89–49.74)	0.07	0.90 (0.48–1.69)	0.73
Medium/Stable (4,682, 32.0%)	22.47 (3.10–163.12)	0.002	1.30 (0.70–2.39)	0.41
Medium low/Decline (2,984, 20.4%)	58.76 (8.10–426.17)	<0.001	1.49 (0.79–2.80)	0.22
Low/Decline (977, 6.7%)	172.29 (23.55–1260.50)	<0.001	1.48 (0.72–3.02)	0.29
**3MS (** ***n*** **, %)**				
High/Stable (9,573, 65.3%)	Reference		Reference	
Medium/Stable (4,137, 28.2%)	3.42 (2.48–4.72)	<0.001	1.28 (0.96–1.71)	0.09
Low/Decline (945, 6.5%)	13.61 (9.56–19.37)	<0.001	1.02 (0.62–1.70)	0.93
**COWAT-F (** ***n*** **, %)**				
High/Increase (785, 5.4%)	Reference		Reference	
Medium high/Increase (3,905, 26.7%)	1.47 (0.62–3.45)	0.38	1.31 (0.63–2.74)	0.47
Medium low/Increase (6,554, 44.7%)	2.59 (1.13–5.91)	0.03	1.49 (0.73–3.04)	0.28
Low/Increase (3,411, 23.3%)	2.41 (1.03–5.63)	0.04	1.30 (0.62–2.73)	0.49
**HVLT-R delayed recall (** ***n*** **, %)**				
High/Increase (3,414, 23.0%)	Reference		Reference	
Medium high/Increase (5,665, 38.7%)	3.30 (1.47–7.42)	0.004	1.23 (0.83–1.82)	0.31
Medium low/Stable (4,320, 29.5%)	12.85 (5.94–27.83)	<0.001	1.25 (0.83–1.88)	0.28
Low/Decline (1,256, 8.6%)	37.73 (17.23-82.64)	<0.001	1.20 (0.72-2.02)	0.49
**SDMT (** ***n*** **, %)**				
High/Decline (862, 5.9%)	Reference		Reference	
Medium high/Decline (3,679, 25.1%)	4.28 (0.57–32.06)	0.16	1.01 (0.41–2.46)	0.99
Medium/Decline (4,868, 33.2%)	9.67 (1.33–70.03)	0.03	1.20 (0.51–2.83)	0.67
Medium low/Decline (3,760, 25.7%)	20.97 (2.90–151.43)	0.003	1.64 (0.70–3.86)	0.26
Low/Decline (1,486, 10.1%)	33.54 (4.59–245.09)	0.001	2.39 (0.99–5.77)	0.05

*3MS, Modified Mini-Mental State Examination; COWAT-F, Controlled Oral Word Association Test-F; HVLT-R, Hopkins Verbal Learning Test–Revised (delayed recall); SDMT, Symbol Digit Modalities Test; OR, odds ratio; CI, confidence interval*.

a *The models adjusted for age (continuous), gender (men; women), ethnicity (Australian white; US white; Hispanic/Latino; Black; other), education (≤12 years; >12 years), smoking status (never; former; current), alcohol intake (never; former; current), living situation (at home alone; at home with someone or in a residential home), body mass index (underweight or normal; overweight; obese), hypertension (yes; no), diabetes (yes; no), dyslipidemia (yes; no), depression (yes; no), weak grip strength [yes; no, defined as grip strength in the lowest 20% at baseline adjusted by gender and body mass index (33), only for incident dementia], slow gait speed (yes; no, defined as gait speed in the lowest 20% at baseline adjusted by gender and height, only for incident dementia), and frailty index [continuous (32)] at the last visit with available data*.

b *Dementia was diagnosed according to the criteria of the Diagnostic and Statistical Manual of Mental Disorders, fourth edition (17)*.

c *Persistent physical disability was defined as being unable to perform or having severe difficulty in performing at least one basic activity of daily living for at least 6 months (17)*.

d *Overall score was defined as the sum of the z-scores of 3MS, COWAT-F, HVLT-R (delayed recall) and SDMT*.

Results regarding death and the composite endpoint are presented in Table 3. The association with increased mortality risk was seen in worse performers of the overall score and the 3MS test, as well as the lowest class of HVLT-R delayed recall – the Low/Decline trajectory (OR: 1.61, 95% CI: 1.09–2.36, *p* = 0.02). For the composite endpoint, associations were observed in the trajectories of all cognitive tests, except COWAT-F.

**Table 3:** Fully adjusted ^a^ associations of cognitive trajectories with incident all-cause mortality and the composite endpoint (*n* = 14,655).

	**All-cause mortality^b^**		**Composite endpoint^c^**	
	**OR (95% CI)**	***P*-value**	**OR (95% CI)**	***P*-value**
**Overall score^d^** **(*****n*****, %)**				
High/Increase (1,454, 9.9%)	Reference		Reference	
Medium high/Increase (4,558, 31.0%)	1.56 (0.88–2.77)	0.13	1.44 (0.95–2.18)	0.08
Medium/Stable (4,682, 32.0%)	1.85 (1.04–3.27)	0.04	2.22 (1.48–3.33)	<0.001
Medium low/Decline (2,984, 20.4%)	1.91 (1.06–3.44)	0.03	3.18 (2.10–4.80)	<0.001
Low/Decline (977, 6.7%)	2.84 (1.52–5.30)	0.001	5.60 (3.61–8.68)	<0.001
**3MS (** ***n*** **, %)**				
High/Stable (9,573, 65.3%)	Reference		Reference	
Medium/Stable (4,137, 28.2%)	1.37 (1.08–1.73)	0.008	1.74 (1.47–2.04)	<0.001
Low/Decline (945, 6.5%)	1.80 (1.28–2.52)	0.001	3.26 (2.60–4.08)	<0.001
**COWAT-F (** ***n*** **, %)**				
High/Increase (785, 5.4%)	Reference		Reference	
Medium high/Increase (3,905, 26.7%)	0.79 (0.48–1.31)	0.36	1.00 (0.69–1.49)	0.96
Medium low/Increase (6,554, 44.7%)	0.83 (0.51–1.34)	0.44	1.25 (0.86–1.82)	0.24
Low/Increase (3,411, 23.3%)	1.12 (0.68–1.84)	0.66	1.37 (0.93–2.02)	0.11
**HVLT-R delayed recall (** ***n*** **, %)**				
High/Increase (3,414, 23.0%)	Reference		Reference	
Medium high/Increase (5,665, 38.7%)	1.01 (0.73–1.39)	0.97	1.23 (0.97–1.57)	0.09
Medium low/Stable (4,320, 29.5%)	1.31 (0.95–1.81)	0.11	1.94 (1.53–2.46)	<0.001
Low/Decline (1,256, 8.6%)	1.61 (1.09–2.36)	0.02	3.40 (2.59–4.45)	<0.001
**SDMT (** ***n*** **, %)**				
High/Decline (862, 5.9%)	Reference		Reference	
Medium high/Decline (3,679, 25.1%)	0.88 (0.47–1.66)	0.70	1.16 (0.70–1.94)	0.56
Medium/Decline (4,868, 33.2%)	1.11 (0.60–2.03)	0.74	1.62 (0.99–2.65)	0.06
Medium low/Decline (3,760, 25.7%)	1.04 (0.56–1.94)	0.89	2.17 (1.32–3.55)	0.002
Low/Decline (1,486, 10.1%)	1.30 (0.68–2.48)	0.43	3.00 (1.80–5.00)	<0.001

*3MS, Modified Mini-Mental State Examination; COWAT-F, Controlled Oral Word Association Test-F; HVLT-R, Hopkins Verbal Learning Test–Revised (delayed recall); SDMT, Symbol Digit Modalities Test; OR, odds ratio; CI, confidence interval*.

a *The models adjusted for age (continuous), gender (men; women), ethnicity (Australian white; US white; Hispanic/Latino; Black; other), education (≤12 years; >12 years), smoking status (never; former; current), alcohol intake (never; former; current), living situation (at home alone; at home with someone or in a residential home), body mass index (underweight or normal; overweight; obese), hypertension (yes; no), diabetes (yes; no), dyslipidemia (yes; no), depression (yes; no), weak grip strength [yes; no, defined as grip strength in the lowest 20% at baseline adjusted by gender and body mass index (33)], slow gait speed [yes; no, defined as gait speed in the lowest 20% at baseline adjusted by gender and height (33)], and frailty index [continuous (32)] at the last visit with complete data*.

b *Death was confirmed with at least two independent sources (e.g., family, or clinical record, or public death notice) (17)*.

c *Composite endpoint was defined as the first occurrence of death, or persistent physical disability, or dementia (17)*.

d *Overall score was defined as the sum of the z-scores of 3MS, COWAT-F, HVLT-R (delayed recall) and SDMT*.

The results from the minimally adjusted models that only included age, gender, ethnicity and education (Supplementary Tables 6, 7) are not materially different from those produced by the fully adjusted models.

## Discussion

Among more than 16,000 community-dwelling older adults initially without dementia or ADL physical disability, we found that low cognitive performers had an increased risk of subsequent geriatric outcomes, compared to those who maintained high cognitive performance. The trajectories of all cognitive tests were associated with incident dementia, but this was less marked for verbal fluency. Death was predicted by low performance in global cognition and episodic memory, while persistent physical disability was only predicted by slow psychomotor speed. These findings suggest that cognitive aging trajectories reflect multiple aspects of health status in late life and could be used as early indicators of risk for major health events, beyond dementia.

Strengths of this study include the large sample size, as well as repeated cognitive assessments that enabled trajectory modeling across 6 years. More importantly, this is the first study to our knowledge that examines the trajectory profiles of multiple cognitive tests using a data-driven approach. This allows a robust comparison across cognitive domains for their associations with important geriatric outcomes and therefore helps to inform targeted strategies for healthy aging. In addition, we visualized the developmental patterns of cognitive changes on a timeframe along with detailed trajectory statistics for each distinct subgroup, which highlights the continuous and dynamic traits of cognitive aging. This longitudinal approach shifts the focus from cognitive function at a single point in time, to cognitive changes (i.e., maintenance, improvement, and decline) over a certain period. In this way, the developmental patterns of the suboptimal trajectories may help to inform when, to whom, and to what extent preventative strategies should be implemented.

We observed an association between low or declining cognitive trajectories in later life and incident dementia. This is in line with well-established evidence that decline may appear in various cognitive domains at the pre-clinical stage of dementia, many years before the diagnosis (34, 35). One study using a similar analytical approach also observed lower levels of hippocampal volume and entorhinal cortical thickness, as well as higher risk of dementia in those with declining memory trajectories (7). Our results showed that trajectories of global cognition, episodic memory and psychomotor speed were all strongly predictive of incident dementia, with an apparent dose-response relationship across the trajectory classes. In contrast, the difference in dementia risk was less marked in the trajectories of COWAT-F, with smaller effect sizes and no dose-response relationship as was seen with other cognitive tests. Interestingly, most participants showed improvements in verbal fluency over time, including those in the lowest COWAT-F trajectory, which possibly reflects practice effects. Indeed, verbal abilities, especially vocabulary, have been known to remain stable or gradually improve with age (4), and are less impacted by the neuropathology of dementia than other cognitive abilities (36). However, previous studies observed poorer verbal fluency in dementia patients compared to the controls (37, 38). Also, the lowest two trajectories of COWAT-F in this study predicted higher dementia risk. Therefore, assessments of verbal abilities may still add value to a comprehensive cognitive battery in dementia screening and prediction, although COWAT-F may be less reflective of brain aging than tests for other cognitive domains.

Although the interdependence between cognitive and physical functioning has been documented (39), evidence regarding how cognitive trajectories could predict future risk of physical disability remains scarce. In a study of 754 community-dwelling older adults with five trajectories of global cognition, the burden of physical disability increased incrementally with the degree of cognitive decline (9). We did not observe a significant association between global cognitive trajectories and physical disability, nor did we find an association in verbal fluency or episodic memory. In the latter two cases, there was a non-significant trend for increasing risk with worse trajectories. It is worth noting that we focused on persistent dysfunction in the same ADL item for over 6 months, which is more stringent than incidental disability investigated in the study of Han and colleagues (9). We did, however, find that the lowest trajectory of psychomotor speed elevated the risk of physical disability by over 2 folds. These results suggest that slowed psychomotor speed may reflect underlying deficits of physical function, although moderate slowing seems to be considerably prevalent in this study. Psychomotor speed may be a central marker of cognitive aging, as it is essential for successful execution of various mental activities (40). This is corroborated by the evidence that psychomotor speed is closely linked to memory and spatial perception, as well as sensory and motor function (39, 41, 42), which are all indispensable in basic daily activities. Therefore, psychomotor speed may be a more sensitive indicator for the future risk of physical disability than the other cognitive domains.

Prior investigation on the extent that cognitive trajectories impact subsequent mortality risk is limited. Four studies found increased mortality risk in those with greater decline in global cognition (10, 11). However, two of these classified participants based on thresholds (e.g., quintile) of cognitive change, which may not adequately reflect the population variance, and the other two were on specific population subgroups (15, 43). Our findings align with previous studies regarding global cognition and add new evidence about other cognitive domains. The worst performers in global cognition and episodic memory showed substantially higher risk of death, and weak associations were also observed in the lower trajectories of psychomotor speed. This suggests that low performance in these cognitive domains may provide a multi-dimensional implication for the aging process, beyond a reflection of neurodegeneration. Trajectories of verbal fluency were not significantly associated with risk of death, which is probably because phonemic fluency and vocabulary are crystalized abilities that are generally acquired via prior learning experiences and are relatively resilient to aging (4). However, we cannot rule out the possibility that observable impairment in verbal abilities may indicate severe disease in some cases.

Terminal decline usually begins several years before death, but the reason remains contentious (44, 45). One explanation is that cognitive decline may be a part of the biological compromise caused by undiagnosed diseases preceding death (44). For example, the decedents with underlying vascular conditions are more likely to experience severe neurological deficits than those without (46). Our findings suggest that cognitive decline can be slow and steady after the age of 70 in the absence of observable cognitive or ADLs dysfunction, even in those at greater risk of impending death. However, their cognitive trajectories, especially in global cognition and episodic memory, may already start to diverge from those of others several years before death.

Our findings highlight the value of strategies to promote cognitive function to help prevent geriatric diseases. To date, several multidomain strategies have proved effective in maintaining cognitive functioning for older individuals in real-world settings. These strategies covered a wide range of domains including but not limited to cognitive training, physical activity, dietary modification and vascular health monitoring (47, 48). In addition, regular interaction with professionals to adjust and optimize cognitive strategies in a timely manner is also essential (47, 49). Therefore, promotional strategies for cognitive function in older age should be advocated broadly. Further, the heterogeneity seen in cognitive aging also suggests the importance of personalized strategies. The early identification of those at risk of adverse outcomes based on their cognitive performance provides an opportunity to formulate preventive strategies at a pre-clinical stage. These should involve not only cognitive training interventions chosen according to cognitive trajectories, but also precision approaches specifically tailored based on the risk profile, health status, and genetic architecture of an individual with declining cognitive trajectories (50).

There are limitations to be acknowledged. First, the ASPREE study only recruited generally healthy participants, and those who died, developed dementia or ADL disability during the trial period were further excluded from the current analysis (to minimize reverse causation during the period when cognitive trajectories were assessed). This likely resulted in a study sample that is healthier than the general older population, and the heterogeneity of cognitive aging may have been underestimated with fewer classes. Therefore, the findings are probably not entirely generalizable to the wider community. Second, the trajectories showed cognitive changes within a specific timeframe, with most participants aged over 70 years. It remains unclear how these outcomes are predicted by cognitive trajectories over a longer period. Further investigation into this area will help characterize cognitive aging beyond the current timeframe and provide information for older adults from more diverse age groups.

## Conclusion

Late-life cognitive trajectories are associated with incident geriatric outcomes, but these vary across cognitive domains. Decline across all cognitive tests was associated with increased risk of dementia. Global cognition and episodic memory were associated with the risk of mortality, while only slow psychomotor speed predicted incident physical disability. These findings indicate that cognitive trajectories are an important predictive marker for neurodegeneration, and the overall health status in older individuals. Therefore, regular global assessments covering multiple cognitive domains in either community routine care or clinical practice may provide important opportunities for early interventions to prevent major health events.

## Data Availability Statement

The original contributions presented in the study are included in the article/Supplementary Material, further inquiries can be directed to the corresponding author/s.

## Ethics Statement

The ASPREE clinical trial was conducted according to the guidelines of the International Conference on Harmonization Good Clinical Practice. The participants provided written informed consent to participate in the trial. The current study was approved by the Monash University Human Research Ethics Committee.

## Author Contributions

Study concept and design: JR, ZW, RLW, and TC. Trial design and Acquisition of data: JM, AM, RLW, RS, RW, ES, and SO. Analysis of data: ZW. Drafting of the manuscript: ZW and JR. Approval of the final version to be submitted, critically revising and editing the manuscript, and interpretation of data: All authors.

## Funding

This work was supported by the National Institute on Aging and the National Cancer Institute at the National Institutes of Health (grant numbers U01AG029824 and U19AG062682); and the National Health and Medical Research Council (NHMRC) of Australia (grant numbers 334047 and 1127060); and Monash University (Australia); and the Victorian Cancer Agency (Australia). JR was supported by an NHMRC Dementia Research Leader Fellowship (grant number APP1135727). ZW is a recipient of the RTP scholarship awarded by Monash University and the Australian Government. The funders had no role in study design and collection, analysis, and interpretation of the results.

## Conflict of Interest

AM reports receiving consulting fees from Alkahest, Inc. and grants from the National Institute on Aging. RS reports grants for clinical research regarding dementia and Alzheimer's disease from the National Institutes of Health, the Centers for Medicare and Medicaid Services, the Department of Defense, and the Illinois Department of Public Health; being as a non-compensated member of the Board of Directors of the Alzheimer's Association–Illinois Chapter; and being as a site principal investigator or sub-investigator for clinical trials and research studies for which his institution (Rush University Medical Center) is sponsored (Amylyx Pharmaceuticals, Inc., Eli Lilly &amp; Co., Inc., Genentech, Inc., Lundbeck, Inc., Merck &amp; Co, Inc., Navidea Biopharmaceuticals, Novartis Pharmaceuticals, Inc., Roche Holdings AG, and Takeda Development Center Americas, Inc.). The remaining authors declare that the research was conducted in the absence of any commercial or financial relationships that could be construed as a potential conflict of interest.

## Publisher's Note

All claims expressed in this article are solely those of the authors and do not necessarily represent those of their affiliated organizations, or those of the publisher, the editors and the reviewers. Any product that may be evaluated in this article, or claim that may be made by its manufacturer, is not guaranteed or endorsed by the publisher.
