# Supplementary material for: Cognitive Trajectories in Community-Dwelling Older Adults and Incident Dementia, Disability and Death: A 10-Year Longitudinal Study
Source: Front Med (Lausanne). 2022 Jun 27;9:917254. doi: 10.3389/fmed.2022.917254 (PMC9271785; doi:10.3389/fmed.2022.917254)
Supplement: Supplementary file 1 [file Data_Sheet_1.docx]

**Supplementary methods**

Model selection criteria (1, 2): 1) model fit (assessed by Akaike information criterion, Bayesian information criterion, and adjusted Bayesian information criterion); 2) average posterior probability ≥0.7 for all classes; 3) odds of correct classification ≥0.5 for all classes; 4) a minimum of 5% of the individuals was assigned to each class; 5) a reasonably narrow confidence interval for each trajectory; 6) close correspondence between estimated probabilities and the percentage of participants assigned to a class.

**Supplementary Table 1. Process of model selection and fit of the selected trajectory model (N=16,174) ^a^**

|  | **AIC** | **BIC** | **Adjusted BIC** | **Entropy** |
| --- | --- | --- | --- | --- |
| Overall score ^b^ |  |  |  |  |
| 1-class model | -135338.32 | -135351.68 | -135349.85 | / |
| 2-class model | -122026.53 | -122053.25 | -122049.61 | 0.84 |
| 3-class model | -116125.55 | -116165.63 | -116160.16 | 0.84 |
| 4-class model | -113364.09 | -113417.53 | -113410.24 | 0.82 |
| 5-class model ^c^ | -111962.01 | -112028.81 | -112019.70 | 0.80 |
| 6-class model ^d^ | -111101.89 | -111182.05 | -111171.11 | 0.78 |
|  |  |  |  |  |
| 3MS |  |  |  |  |
| 1-class model | -158650.71 | -158664.10 | -158662.25 | / |
| 2-class model | -149528.44 | -149555.21 | -149551.52 | 0.87 |
| 3-class model ^c^ | -146822.84 | -146862.99 | -146857.45 | 0.81 |
| 4-class model ^d^ | -146005.94 | -146059.47 | -146052.08 | 0.75 |
|  |  |  |  |  |
| COWAT-F |  |  |  |  |
| 1-class model | -165243.01 | -165256.39 | -165254.54 | / |
| 2-class model | -156688.46 | -156715.22 | -156711.53 | 0.77 |
| 3-class model | -153787.15 | -153827.29 | -153821.76 | 0.76 |
| 4-class model ^c^ | -152730.64 | -152784.16 | -152776.78 | 0.73 |
| 5-class model ^d^ | -152281.27 | -152348.17 | -152338.95 | 0.72 |
|  |  |  |  |  |
| HVLT-R |  |  |  |  |
| 1-class model | -131093.09 | -131106.46 | -131104.63 | / |
| 2-class model | -120995.75 | -121022.49 | -121018.82 | 0.79 |
| 3-class model | -117559.64 | -117599.74 | -117594.25 | 0.78 |
| 4-class model ^c^ | -116327.91 | -116381.38 | -116374.06 | 0.75 |
| 5-class model ^d^ | -115911.48 | -115978.32 | -115969.16 | 0.72 |
|  |  |  |  |  |
| SDMT |  |  |  |  |
| 1-class model | -204050.00 | -204063.37 | -204061.53 | / |
| 2-class model | -192093.44 | -192120.18 | -192116.51 | 0.82 |
| 3-class model | -187410.76 | -187450.88 | -187445.37 | 0.81 |
| 4-class model | -185541.31 | -185594.80 | -185587.45 | 0.79 |
| 5-class model ^c^ | -184731.51 | -184798.38 | -184789.20 | 0.77 |
| 6-class model ^d^ | -184361.30 | -184441.54 | -184430.52 | 0.74 |

Abbreviations: AIC, Akaike information criterion; BIC, Bayesian information criterion; 3MS, Modified Mini-Mental State Examination; COWAT-F, Controlled Oral Word Association Test-F; HVLT-R, Hopkins Verbal Learning Test–Revised (delayed recall); SDMT, Symbol Digit Modalities Test

^a^ Group-based trajectory modelling only included 1) those with available data of all the four cognitive tests at baseline and at least one subsequent timepoint; and 2) those who did not reach the endpoints of this study during the trial period, thus a number of 16,174 participants were eligible.

^b^ Overall score was defined as the sum of the z-scores of 3MS, COWAT, HVLT-R (delayed recall) and SDMT.

^c^ The model was selected.

^d^ The model was rejected based on one or more model selection criteria.

**Supplementary Table 2. Assessment of adequacy of the selected trajectory model (N=16,174)**

|  | **No. (%)** | **AvePP (%)** | **OCC** | **EP (%)** |
| --- | --- | --- | --- | --- |
| Overall score ^a^ |  |  |  |  |
| High/Increase | 1,542 (9.5) | 87.8 | 76.3 | 8.6 |
| Medium high/Increase | 4,891 (30.2) | 86.3 | 18.6 | 25.2 |
| Medium/Stable | 5,130 (31.7) | 86.0 | 16.9 | 26.6 |
| Medium low/Decline | 3,422 (21.2) | 88.2 | 34.2 | 17.9 |
| Low/Decline | 1,189 (7.4) | 91.6 | 162.3 | 6.3 |
|  |  |  |  |  |
| 3MS |  |  |  |  |
| High/Stable | 10,371 (64.1) | 93.3 | 12.2 | 53.2 |
| Medium/Stable | 4,644 (28.7) | 86.6 | 19.2 | 25.2 |
| Low/Decline | 1,159 (7.2) | 92.0 | 171.3 | 6.2 |
|  |  |  |  |  |
| COWAT-F |  |  |  |  |
| High/Increase | 829 (5.1) | 87.8 | 148.3 | 4.6 |
| Medium high/Increase | 4,251 (26.3) | 85.0 | 19.3 | 22.8 |
| Medium low/Increase | 7,248 (44.8) | 81.9 | 7.9 | 36.5 |
| Low/Increase | 3,846 (23.8) | 85.6 | 22.6 | 20.8 |
|  |  |  |  |  |
| HVLT-R |  |  |  |  |
| High/Increase | 3,618 (22.4) | 88.7 | 32.5 | 19.5 |
| Medium high/Increase | 6,171 (38.1) | 82.9 | 10.5 | 31.6 |
| Medium low/Stable | 4,904 (30.3) | 83.8 | 15.3 | 25.3 |
| Low/Decline | 1,481 (9.2) | 87.5 | 78.0 | 8.2 |
|  |  |  |  |  |
| SDMT |  |  |  |  |
| High/Decline | 927 (5.7) | 85.5 | 107.6 | 5.2 |
| Medium high/Decline | 3,978 (24.6) | 85.0 | 21.8 | 20.6 |
| Medium/Decline | 5,317 (32.9) | 83.4 | 13.3 | 27.4 |
| Medium low/Decline | 4,205 (26.0) | 84.5 | 19.3 | 22.0 |
| Low/Decline | 1,747 (10.8) | 88.7 | 75.3 | 9.4 |

Abbreviations: AvePP, average posterior probability; OCC, odds of correct classification; EP, estimated probability; 3MS, Modified Mini-Mental State Examination; COWAT-F, Controlled Oral Word Association Test-F; HVLT-R, Hopkins Verbal Learning Test–Revised (delayed recall); SDMT, Symbol Digit Modalities Test

^a^ Overall score was defined as the sum of the z-scores of 3MS, COWAT-F, HVLT-R (delayed recall) and SDMT.

**Supplementary Table 3. Number of timepoints with complete cognitive data by cognitive trajectory subgroup (N=16,174)**

| **Cognitive test** | **Number of cognitive assessments, Median (inter-quartile range)** | | | | |
| --- | --- | --- | --- | --- | --- |
|  | Class 1 | Class 2 | Class 3 | Class 4 | Class 5 |
| Overall score ^a^ | 4 (3-4) | 4 (3-4) | 3 (3-4) | 3 (3-4) | 3 (2-4) |
| 3MS | 4 (3-4) | 3 (3-4) | 3 (3-4) | / | / |
| COWAT-F | 4 (3-4) | 4 (3-4) | 3 (3-4) | 3 (3-4) | / |
| HVLT-R delayed recall | 4 (3-4) | 3 (3-4) | 3 (3-4) | 3 (3-4) | / |
| SDMT | 4 (3-4) | 3 (3-4) | 3 (3-4) | 3 (3-4) | 3 (3-4) |

Abbreviations: 3MS, Modified Mini-Mental State Examination; COWAT-F, Controlled Oral Word Association Test-F; HVLT-R, Hopkins Verbal Learning Test–Revised (delayed recall); SDMT, Symbol Digit Modalities Test

^a^ Overall score was defined as the sum of the z-scores of 3MS, COWAT-F, HVLT-R (delayed recall) and SDMT.

**Supplementary Table 4. Intercept and slope parameters of the identified cognitive trajectory subgroups (N=16,174)**

|  | **Class 1** | **Class 2** | **Class 3** | **Class 4** | **Class 5** |
| --- | --- | --- | --- | --- | --- |
| Overall score ^a^ |  |  |  |  |  |
| Intercept | 4.50 | 2.39 | 0.28 | -1.97 | -4.62 |
| Slope | 0.13 | 0.10 | 0.04 | -0.06 | -0.18 |
| 3MS |  |  |  |  |  |
| Intercept | 96.40 | 91.68 | 85.54 | / | / |
| Slope | 0.12 | -0.02 | -0.38 | / | / |
| COWAT-F |  |  |  |  |  |
| Intercept | 20.91 | 16.23 | 11.90 | 7.99 | / |
| Slope | 0.68 | 0.28 | 0.24 | 0.14 | / |
| HVLT-R delayed recall |  |  |  |  |  |
| Intercept | 11.63 | 8.92 | 6.40 | 3.57 | / |
| Slope | 0.23 | 0.14 | -0.05 | -0.27 | / |
| SDMT |  |  |  |  |  |
| Intercept | 54.11 | 46.32 | 38.73 | 31.06 | 22.63 |
| Slope | -0.31 | -0.41 | -0.50 | -0.52 | -0.47 |

Abbreviations: 3MS, Modified Mini-Mental State Examination; COWAT-F, Controlled Oral Word Association Test-F; HVLT-R, Hopkins Verbal Learning Test–Revised (delayed recall); SDMT, Symbol Digit Modalities Test

^a^ Overall score was defined as the sum of the z-scores of 3MS, COWAT-F, HVLT-R (delayed recall) and SDMT.

**Supplementary Table 5. Comparison of basic characteristics between participants included in and excluded from the association analysis (n=19,114)**

| **Baseline characteristics** | **Included participants**  **(n=14,655, 76.7%)** | **Excluded participants**  **(4,459, 23.3%)** | **P-value ^a^** |
| --- | --- | --- | --- |
| Age, years |  |  | <0.001 |
| 65-69 ^b^ | 81 (0.6) | 153 (3.4) |  |
| 70-74 | 2,461 (16.8) | 646 (14.5) |  |
| 75-79 | 7,124 (48.6) | 1,616 (36.2) |  |
| 80-84 | 3,390 (23.1) | 1,083 (24.3) |  |
| ≥85 | 1,599 (10.9) | 961 (21.6) |  |
| Gender |  |  | 0.05 |
| Men | 6,446 (44.0) | 1,886 (42.3) |  |
| Women | 8,209 (56.0) | 2,573 (57.7) |  |
| Ethnicity |  |  | <0.001 |
| Australian white | 13,009 (88.8) | 3,353 (75.2) |  |
| US white | 709 (4.8) | 379 (8.5) |  |
| African American | 443 (3.0) | 458 (10.3) |  |
| Hispanic/Latino | 299 (2.0) | 189 (4.2) |  |
| Other | 195 (1.3) | 80 (1.8) |  |
| Education, years |  |  | 0.008 |
| <12 | 8,323 (56.8) | 2,632 (59.0) |  |
| ≥12 | 6,332 (43.2) | 1,826 (41.0) |  |
| Missing | 0 (0.0) | 1 (0.0) |  |
| Living situation |  |  | 0.27 |
| Alone at home | 5,137 (35.1) | 1,603 (36.0) |  |
| With someone | 9,518 (64.9) | 2,856 (64.0) |  |
| Smoking status |  |  | <0.001 |
| Current | 355 (2.4) | 262 (5.9) |  |
| Former | 6,018 (41.1) | 1,906 (42.8) |  |
| Never | 8,282 (56.5) | 2,291 (51.4) |  |
| Alcohol intake |  |  | <0.001 |
| Current-high risk | 3,750 (25.6) | 937 (21.0) |  |
| Current-low risk | 6,565 (44.8) | 1,742 (39.1) |  |
| Former | 2,161 (14.8) | 892 (20.0) |  |
| Never | 2,179 (14.9) | 888 (19.9) |  |
| Hypertension ^c^ |  |  | <0.001 |
| Yes | 11,187 (76.3) | 3,529 (79.1) |  |
| No | 3,468 (23.7) | 930 (20.9) |  |
| Diabetes mellitus ^d^ |  |  | <0.001 |
| Yes | 1,480 (10.1) | 627 (14.1) |  |
| No | 13,175 (89.9) | 3,832 (85.9) |  |
| Dyslipidaemia ^e^ |  |  | 0.003 |
| Yes | 9,516 (64.9) | 2,787 (62.5) |  |
| No | 5,139 (35.1) | 1,672 (37.5) |  |
| Depression ^f^ |  |  | <0.001 |
| Yes | 2,659 (18.1) | 966 (21.7) |  |
| No | 11,996 (81.9) | 3,492 (78.3) |  |
| Missing | 0 (0.0) | 0 (0.0) |  |
| Weak grip strength ^g^ |  |  | <0.001 |
| Yes | 3,848 (26.3) | 1,533 (34.4) |  |
| No | 10,807 (73.7) | 2,864 (64.2) |  |
| Missing | 0 (0.0) | 62 (1.4) |  |
| Slow gait speed ^g^ |  |  | <0.001 |
| Yes | 3,850 (26.3) | 1,288 (28.9) |  |
| No | 10,805 (73.7) | 1,744 (39.1) |  |
| Missing | 0 (0.0) | 1,427 (32.0) |  |
| Body mass index ^h^ |  |  | <0.001 |
| Underweight/Normal | 4,335 (29.6) | 1,462 (32.8) |  |
| Overweight | 6,455 (44.1) | 1,743 (39.1) |  |
| Obese | 3,865 (26.4) | 1,216 (27.3) |  |
| Missing | 0 (0.0) | 38 (0.9) |  |

^a^ P-values are based on Pearson’s chi-squared test or Fisher’s exact test.

^b^ Only includes U.S. African American or Hispanic/Latino participants, who were eligible to enroll from 65 years or above (all other participants needed to be 70 years or above to be recruited).

^c^ Hypertension was defined as on treatment for high BP or BP >140/90 mmHg at study entry.

^d^ Diabetes was defined from self-report or fasting glucose ≥126mg/dL (≥7 mmol/L) or on treatment for diabetes.

^e^ Dyslipidemia was defined as those taking cholesterol-lowering medications or serum cholesterol ≥212 mg/dL (≥5 mmol/L; Australia) and ≥240 mg/dL (≥6.2 mmol/L; U.S.) or LDL > 160 mg/dL (>4.1 mmol/L).

^f^ Depression was defined as CES-D-10 ≥8.

^g^ Weak grip strength and slow gait speed were defined using the adapted Fried frailty criteria (3).

^h^ Overweight was defined as body mass index ≥25 kg/m^2^ and obesity as body mass index ≥30 kg/m^2^.

**Supplementary table 6. Minimally adjusted ^a^ associations of cognitive trajectories with incident dementia and persistent physical disability (n=14,655)**

|  | **Dementia ^b^** | | **Persistent physical disability ^c^** | |
| --- | --- | --- | --- | --- |
|  | **OR (95% CI)** | **P-value** | **OR (95% CI)** | **P-value** |
| Overall score ^d^ (n, %) |  |  |  |  |
| High/Increase (1,509, 9.7%) | Reference |  | Reference |  |
| Medium high/Increase (4,770, 30.5%) | 6.55 (0.86-49.03) | 0.07 | 1.09 (0.58-2.03) | 0.79 |
| Medium/Stable (4,983, 31.9%) | 22.21 (3.06-160.95) | 0.002 | 1.80 (0.99-3.29) | 0.06 |
| Medium low/Decline (3,258, 20.8%) | 58.84 (8.14-425.51) | <0.001 | 2.37 (1.28-4.41) | 0.006 |
| Low/Decline (1,124, 7.2%) | 176.29 (24.20-1284.17) | <0.001 | 2.50 (1.24-5.02) | 0.01 |
|  |  |  |  |  |
| 3MS (n, %) |  |  |  |  |
| High/Stable (10,084, 64.5%) | Reference |  | Reference |  |
| Medium/Stable (4,473, 28.6%) | 3.56 (2.59-4.89) | <0.001 | 1.47 (1.12-1.94) | 0.006 |
| Low/Decline (1,087, 7.0%) | 14.18 (10.04-20.03) | <0.001 | 1.11 (0.68-1.83) | 0.67 |
|  |  |  |  |  |
| COWAT-F (n, %) |  |  |  |  |
| High/Increase (808, 5.2%) | Reference |  | Reference |  |
| Medium high/Increase 2 (4,121, 26.3%) | 1.54 (0.66-3.63) | 0.32 | 1.33 (0.66-2.71) | 0.42 |
| Medium low/Increase (7,015, 44.8%) | 2.71 (1.19-6.19) | 0.02 | 1.57 (0.79-3.12) | 0.20 |
| Low/Increase (3,700, 23.7%) | 2.62 (1.12-6.12) | 0.03 | 1.59 (0.78-3.26) | 0.20 |
|  |  |  |  |  |
| HVLT-R delayed recall (n, %) |  |  |  |  |
| High/Increase (3,551, 22.7%) | Reference |  | Reference |  |
| Medium high/Increase 2 (5,981, 38.2%) | 3.35 (1.49-7.52) | 0.004 | 1.40 (0.96-2.06) | 0.08 |
| Medium low/Stable (4,710, 30.1%) | 13.36 (6.18-28.86) | <0.001 | 1.52 (1.02-2.26) | 0.04 |
| Low/Decline (1,402, 9.0%) | 41.74 (19.13-91.10) | <0.001 | 1.56 (0.94-2.57) | 0.09 |
|  |  |  |  |  |
| SDMT (n, %) |  |  |  |  |
| High/Decline (899, 5.8%) | Reference |  | Reference |  |
| Medium high/Decline (3,872, 24.8%) | 4.39 (0.59-32.86) | 0.15 | 1.13 (0.47-2.73) | 0.78 |
| Medium/Decline (5,160, 33.0%) | 10.16 (1.40-73.54) | 0.02 | 1.76 (0.76-4.09) | 0.19 |
| Medium low/Decline (4,040, 25.8%) | 22.65 (3.14-163.34) | 0.003 | 2.66 (1.14-6.20) | 0.02 |
| Low/Decline (1,673, 10.7%) | 39.65 (5.44-288.76) | <0.001 | 4.38 (1.83-10.45) | 0.001 |

Abbreviations: 3MS, Modified Mini-Mental State Examination; COWAT-F, Controlled Oral Word Association Test-F; HVLT-R, Hopkins Verbal Learning Test–Revised (delayed recall); SDMT, Symbol Digit Modalities Test; OR, odds ratio; CI, confidence interval

^a^ The models adjusted for age (continuous), gender (men; women), ethnicity (Australian white; US white; Hispanic/Latino; Black; other) and education (≤12 years; >12 years).

^b^ Dementia was diagnosed according to the criteria of the Diagnostic and Statistical Manual of Mental Disorders, fourth edition (4).

^c^ Persistent physical disability was defined as being unable to perform or having severe difficulty in performing at least one basic activity of daily living for at least 6 months (4).

^d^ Overall score was defined as the sum of the z-scores of 3MS, COWAT-F, HVLT-R (delayed recall) and SDMT.

**Supplementary table 7. Minimally adjusted ^a^ associations of cognitive trajectories with incident all-cause mortality and the composite endpoint (n=14,655)**

|  | **All-cause mortality ^b^** | | **Composite endpoint ^c^** | |
| --- | --- | --- | --- | --- |
|  | **OR (95% CI)** | **P-value** | **OR (95% CI)** | **P-value** |
| Overall score ^d^ (n, %) |  |  |  |  |
| High/Increase (1,509, 9.7%) | Reference |  | Reference |  |
| Medium high/Increase (4,770, 30.5%) | 1.60 (0.90-2.84) | 0.11 | 1.55 (1.03-2.35) | 0.04 |
| Medium/Stable (4,983, 31.9%) | 2.04 (1.16-3.60) | 0.01 | 2.62 (1.75-3.92) | <0.001 |
| Medium low/Decline (3,258, 20.8%) | 2.28 (1.27-4.08) | 0.006 | 4.08 (2.71-6.14) | <0.001 |
| Low/Decline (1,124, 7.2%) | 3.50 (1.89-6.50) | <0.001 | 7.45 (4.84-11.47) | <0.001 |
|  |  |  |  |  |
| 3MS (n, %) |  |  |  |  |
| High/Stable (10,084, 64.5%) | Reference |  | Reference |  |
| Medium/Stable (4,473, 28.6%) | 1.48 (1.18-1.87) | 0.001 | 1.92 (1.63-2.25) | <0.001 |
| Low/Decline (1,087, 7.0%) | 1.93 (1.38-2.69) | <0.001 | 3.50 (2.82-4.36) | <0.001 |
|  |  |  |  |  |
| COWAT-F (n, %) |  |  |  |  |
| High/Increase (808, 5.2%) | Reference |  | Reference |  |
| Medium high/Increase 2 (4,121, 26.3%) | 0.78 (0.47-1.28) | 0.33 | 1.04 (0.71-1.52) | 0.84 |
| Medium low/Increase (7,015, 44.8%) | 0.82 (0.51-1.33) | 0.42 | 1.31 (0.91-1.89) | 0.15 |
| Low/Increase (3,700, 23.7%) | 1.17 (0.72-1.72) | 0.52 | 1.57 (1.07-2.29) | 0.02 |
|  |  |  |  |  |
| HVLT-R delayed recall (n, %) |  |  |  |  |
| High/Increase (3,551, 22.7%) | Reference |  | Reference |  |
| Medium high/Increase 2 (5,981, 38.2%) | 1.07 (0.78-1.48) | 0.66 | 1.35 (1.06-1.71) | 0.01 |
| Medium low/Stable (4,710, 30.1%) | 1.44 (1.04-1.98) | 0.03 | 2.19 (1.74-2.77) | <0.001 |
| Low/Decline (1,402, 9.0%) | 1.83 (1.26-2.67) | 0.002 | 3.97 (3.05-5.17) | <0.001 |
|  |  |  |  |  |
| SDMT (n, %) |  |  |  |  |
| High/Decline (899, 5.8%) | Reference |  | Reference |  |
| Medium high/Decline (3,872, 24.8%) | 0.93 (0.49-1.75) | 0.82 | 1.25 (0.75-2.08) | 0.39 |
| Medium/Decline (5,160, 33.0%) | 1.29 (0.71-2.37) | 0.41 | 1.97 (1.21-3.21) | 0.007 |
| Medium low/Decline (4,040, 25.8%) | 1.30 (0.70-2.40) | 0.40 | 2.87 (1.76-4.69) | <0.001 |
| Low/Decline (1,673, 10.7%) | 1.77 (0.94-3.37) | 0.08 | 4.45 (2.69-7.38) | <0.001 |

Abbreviations: 3MS, Modified Mini-Mental State Examination; COWAT-F, Controlled Oral Word Association Test-F; HVLT-R, Hopkins Verbal Learning Test–Revised (delayed recall); SDMT, Symbol Digit Modalities Test; OR, odds ratio; CI, confidence interval

^a^ The models adjusted for age (continuous), gender (men; women), ethnicity (Australian white; US white; Hispanic/Latino; Black; other) and education (≤12 years; >12 years).

^b^ Death was confirmed with at least two independent sources (e.g., family, or clinical record, or public death notice) (4).

^c^ Composite endpoint was defined as the first occurrence of death, or persistent physical disability, or dementia (4).

^d^ Overall score was defined as the sum of the z-scores of 3MS, COWAT-F, HVLT-R (delayed recall) and SDMT.

Reference

1. Andruff H, Carraro N, Thompson A, Gaudreau P, Louvet B. Latent Class Growth Modelling: A Tutorial. Tutorials in Quantitative Methods for Psychology. 2009;5(1):11-24.

2. Nagin DS. Group-based trajectory modeling: an overview. Ann Nutr Metab. 2014;65(2-3):205-10.

3. Fried LP, Tangen CM, Walston J, Newman AB, Hirsch C, Gottdiener J, et al. Frailty in older adults: evidence for a phenotype. J Gerontol A Biol Sci Med Sci. 2001;56(3):M146-56.

4. Wolfe R, Murray AM, Woods RL, Kirpach B, Gilbertson D, Shah RC, et al. The aspirin in reducing events in the elderly trial: Statistical analysis plan. Int J Stroke. 2018;13(3):335-8.
